# Risk Factors Associated With Abnormal Urinalysis in Children

**DOI:** 10.3389/fped.2021.649068

**Published:** 2021-03-25

**Authors:** Xuhui Zhong, Jie Ding, Zheng Wang, Yan Gao, Yubin Wu, Ying Shen, Hongmei Song, Zhengyan Zhao, Xinxin Chen, Puhong Zhang, Guobin Xu, Chen Yao, Hui Zhang, Fu Zhong, Ying Tang, Hui Wang, Wei Wang, Wenhao Li, Wanxia Zhang, Sainan Zhu, Meixia Shang

**Affiliations:** ^1^Department of Pediatrics, Peking University First Hospital, Beijing, China; ^2^Department of Pediatrics, Sichuan University West China Second University Hospital, Chengdu, China; ^3^Department of Pediatric Nephrology, Guangzhou Women and Children's Medical Center, Guangzhou, China; ^4^Department of Pediatrics, Shengjing Hospital of China Medical University, Shenyang, China; ^5^Department of Pediatric Nephrology, Beijing Children's Hospital, Capital Medical University, Beijing, China; ^6^Department of Pediatrics, Peking Union Medical College Hospital, Beijing, China; ^7^Department of Child Healthcare, The Children's Hospital of Zhejiang University School of Medicine, Hangzhou, China; ^8^Department of Infant Healthcare Section, Beijing Obstetrics and Gynecology Hospital, Capital Medical University, Beijing, China; ^9^Diabetes Research Program, The George Institute for Global Health at Peking University Health Science Center, Beijing, China; ^10^Department of Clinical Laboratory, Peking University First Hospital, Beijing, China; ^11^Department of Biostatistics, Peking University First Hospital, Beijing, China

**Keywords:** urinalysis, children, targeted screening, hematuria, proteinuria, leukocyturia

## Abstract

**Background:** Targeted urinalysis has been suggested to improve screening efficiency in adults. However, there is no well-defined target population in children yet, with limited information on the risk factors for urinalysis abnormalities.

**Methods:** Children from infants to 17 years old were randomly selected. Dipstick urinalysis was initially performed. Among those who were abnormal, a repeat dipstick or dipstick with microscopic urinalysis was performed for confirmation.

**Results:** In total, 70,822 children were included, with 37,866 boys and 32,956 girls. Prevalence of abnormal urinalysis was 4.3%. Age was significantly associated with abnormal urinalysis, with the highest prevalence among 12–14-year-olds. Girls were 2.0 times more likely to exhibit abnormalities. Compared with children whose guardians had a college degree or higher, those whose guardians had a high school degree or lower had a higher likelihood of abnormalities. Geographic location was also associated with abnormal results.

**Conclusion:** Girls, children aged 12–14 years old, and children whose guardians had a low educational level and children in certain geographic locations were significantly associated with abnormal urinalysis. Identification of children at high risk would contribute to targeted urinalysis screening.

## Introduction

Chronic kidney disease (CKD) is considered one of the most harmful diseases among children, causing heavy social and economic burdens on patients' families (1, 2). Early detection and prompt intervention are essential to reduce the burdens of CKD. Optional strategies to detect CKD in healthy children include urinalysis, kidney ultrasound and serum markers of kidney function (3–5). Among them, urinalysis has been broadly utilized for CKD screening because it is cheap, widely available and non-invasive.

However, the specificity and cost-effectiveness of urinalysis screening in children have been doubted for decades. Most Asian pediatric nephrologists support routine urinalysis in children. In Japan, Korea and Taiwan, mass urinary screening programs for children have been established for many years (6–9). Japanese experts have indicated that kidney diseases are more responsive to therapy and that progression to kidney failure slows, owing to their school screening program (6). However, American experts hold a quite different opinion. They concluded that urinalysis screening is unnecessary and cost-ineffective in children (10). In 2007, the American Academy of Pediatrics (AAP) even removed screening urine dipsticks from the practice recommendation for well-child care (11). Most of the controversies should be attributed to the low prevalence of abnormalities and low specificity of urinalysis in the screening process (6, 12).

To improve the efficiency of urinalysis screening programs, a high-risk population targeting approach has been suggested in adults with diabetes, hypertension or low socioeconomic status (13, 14). Unfortunately, there is not enough information about target populations among children. In previous reports, urinary abnormalities have been detected to be more common among girls, children at certain ages or children with low socioeconomic status (6, 15, 16). However, a comprehensive investigation among children of all ages is still needed to identify children at high risk.

Therefore, we performed a cross-sectional multicenter survey to investigate the prevalence of abnormal urinalysis among children of all ages. Aiming to reveal risk factors for abnormal urinalysis, this study might provide important clues about target populations in children. It would contribute to targeted urinalysis screening, improving the current screening practice.

## Methods

### Participant Children

From April 2009 to January 2012, participating children were enrolled by stratified, clustered and random sampling. Five cities located in northern, northeastern, eastern, southern and southwestern China were selected. From each city, three administrative districts were sampled using computer-generated random numbers. Then, from each administrative district, eight health care centers, eight kindergartens and eight schools were sampled using the same random procedure. Next, 42 children per year old were randomly enrolled from each health care center, kindergarten or school. For children from 3 to 17 years old, clusters of classes from the kindergartens and schools were randomly selected by computer. Informed consents were obtained from the guardians. The study was approved by the Institutional Review Board in 2009.

### Urine Collection and Urinalysis

The enrolled children were asked to collect clean voided mid-stream morning urine specimens. For children not toilet trained, urine samples were collected with urine bags. Urine samples were gathered and delivered to the laboratory at 4°C within 4 h. For adolescent girls during the menstrual period, urine samples were collected 2 weeks later.

For the first urinalysis test, dipstick urinalysis was performed. Children were defined as abnormal if they tested positive for occult blood, protein, nitrite, glucose, or leukocytes.

Children with abnormal results were notified and asked to collect clean voided midstream morning urine specimens 2 weeks later for the second urinalysis. Dipstick urinalysis was repeated. If the second dipstick urinalysis was abnormal for occult blood, nitrite or leukocytes, microscopic urinalysis was performed for confirmation. Finally, according to the result of the second urinalysis, “abnormal urinalysis” was confirmed as follows: (1) hematuria was defined when the red blood cell count was ≥3/hp by microscopy, (2) leukocyturia was defined by a white blood cell count of ≥5/hp, (3) proteinuria was defined as urinary dipstick protein ranging from trace to 4+, and (4) glycosuria was defined as urinary dipstick glucose ranging from trace to 4+.

### Questionnaire Survey

Guardians of participant children were invited to fill out questionnaires. Demographics, socioeconomic status, prior history of kidney diseases, guardian's awareness of kidney diseases, and attitudes toward routine urinalysis in children were collected. The survey data entered were double-checked. Children with a history of kidney disease were excluded.

Good awareness of kidney diseases was defined as guardians knew about disease symptoms (edema, hematuria, proteinuria, flank pain, or hypertension), complications (growth retardation, anemia), and treatment options (Western medicine, traditional Chinese medicine). Poor awareness was defined if guardians had no idea about the signs of kidney diseases, did not know kidney diseases were harmful to children, considered kidney diseases harmless, knew little about available treatment, considered treatment unnecessary or ineffective, or believed in folk prescriptions.

### Data Analyses

Descriptive statistics were performed as frequencies and percentages for categorical variables or as the means and standard deviations for normally distributed continuous variables. Binary logistic regression was conducted to identify factors associated with abnormal urinalysis results or response to the second urinalysis. We report crude and multivariable-adjusted odds ratios with 95% CIs. Covariates included in the model were sex, age (per year old), guardian's education level (less than high school, high school or technical secondary school, Junior college, college degree or more), geographic location (southwest, east, north, northeast, south region of China), monthly household income (<5,000 CNY, 5,000–10,000 CNY, >10,000 CNY, refused to answer), guardian's awareness of kidney diseases in children (poor, good), guardian's attitude toward routine urinalysis in children (routine urinalysis is necessary, others), and family size (>3 family members, ≤ 3 family members). *P* <0.05 were considered statistically significant. All statistical analyses were conducted using SPSS for Windows, version 19.0 and SAS software version 9.3 (SAS Institute, Cary, NC, USA).

Sample size was calculated using the formula outlined below. The prevalence of an abnormal urinalysis result among children was supposed to be 3.0%, with a precision of 0.7%. The study was designed to enroll children from 17 age groups, i.e., from infants to 17 years old. Unresponsive rate was assumed to be 30%. Given cluster and random sampling (×1.5), the sample size was expected to be 83,110 cases.

$$\begin{array}{l} n=\frac{Z_{\alpha }^{2}p\left(1-p\right)}{d^{2}} \end{array}$$

*n*: sample size

*p:* expected prevalence of an abnormal urinalysis result among children

*d*: precision

α = 0.05

## Results

**Figure 1:**
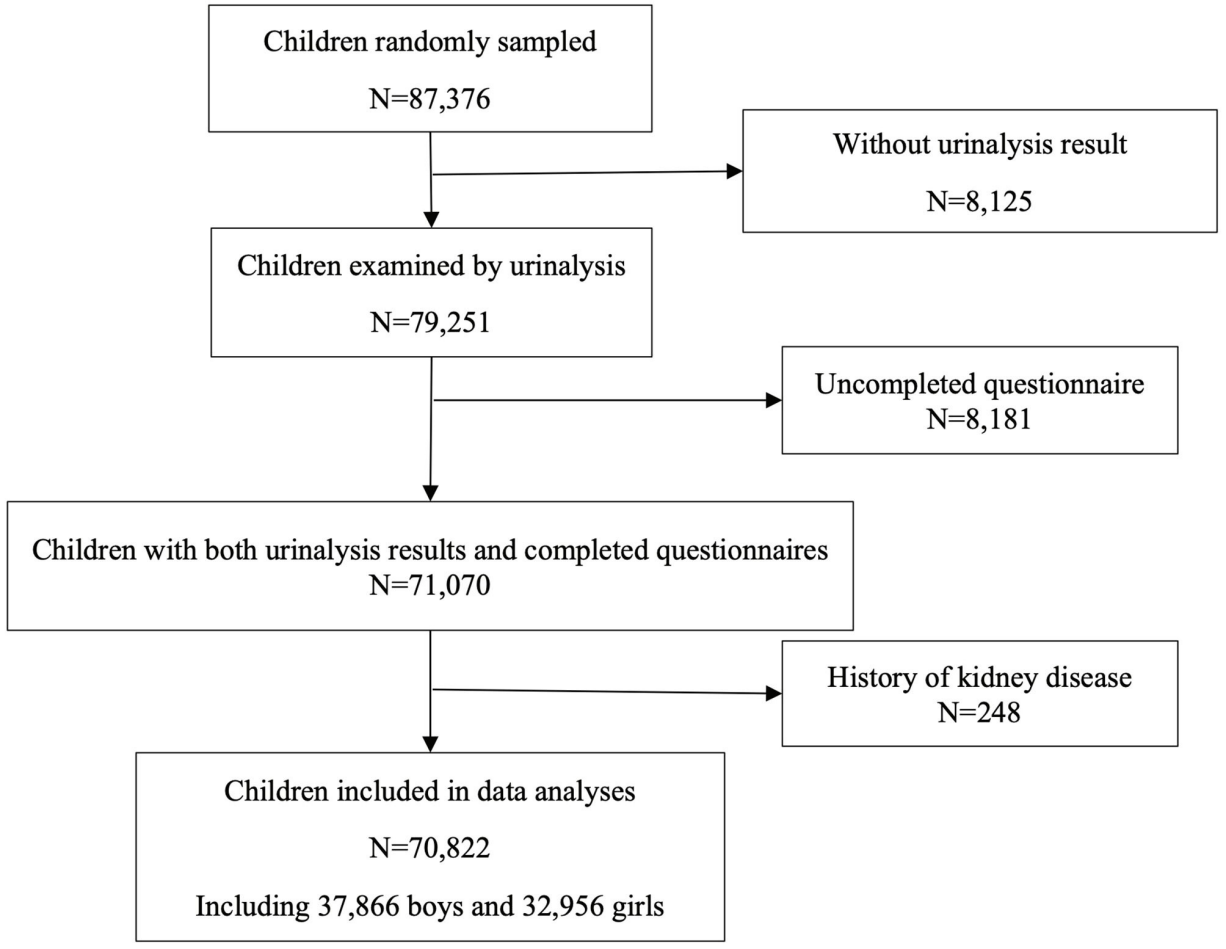
Flow diagram of children included.

At enrollment, 87,376 children were randomly sampled from 5 cities. There were 71,070 children with both urinalysis results and completed questionnaires. Children with a history of kidney disease were excluded. Eventually, 70,822 children were included in the data analyses, with 37,866 boys and 32,956 girls, equally distributed across age groups (Figure 1).

The characteristics of the demographics and socioeconomic status of the participating children are summarized in Table 1.

**Table 1:** Characteristics of demographics and socioeconomic status of children included.

	**Participant children (*n =* 70,822)**
	**No. (%)**
Age, mean (SD), years old	9.0 (5.0)
Boys	37,866 (53.5)
Family size	69,682^a^
≤ 3 family members	43,662 (62.7)^a^
>3 family members	26,020 (37.3)^a^
Geographic location	70,822
Beijing (north)	16,687 (23.6)
Chengdu (southwest)	17,821 (25.2)
Guangzhou (south)	16,526 (23.3)
Shenyang (northeast)	14,768 (20.9)
Hangzhou (east)	5,020 (7.1)
Guardian's education level	69,131^a^
Less than high school	10,576 (15.3)^a^
High school or technical secondary school	15,604 (22.6)^a^
Junior college degree	17,374 (25.1)^a^
College degree or more	25,577 (37.0)^a^
Monthly household income	70,822
<5,000 CNY	26,270 (37.1)
5,000–10,000 CNY	23,903 (33.8)
>10,000 CNY	4,399 (6.2)
Refused to answer	16,250 (22.9)

a *missing values were not included in the percentage calculation*.

### Prevalence of Abnormal Urinalysis

In the first dipstick analysis, abnormal results were observed in 9,891 (14.0%) of the 70,822 children. Positive protein results (5.8%) were most commonly reported, followed by occult blood (4.6%), leukocytes (3.7%), nitrite (1.3%), and glucose (0.3%). For the second urinalysis, 5,438 children responded. Among the responsive children, 30.8% were confirmed to be abnormal. Eventually, the prevalence of abnormal urinalysis results was 4.3%, irrespective of non-response bias. Isolated proteinuria (1.5%) was the most prevalent, followed by isolated hematuria (1.4%), isolated leukocyturia (0.8%), hematuria and proteinuria (0.3%), isolated glycosuria (0.1%), etc.

### Factors Associated With Abnormal Urinalysis Results

**Figure 2:**
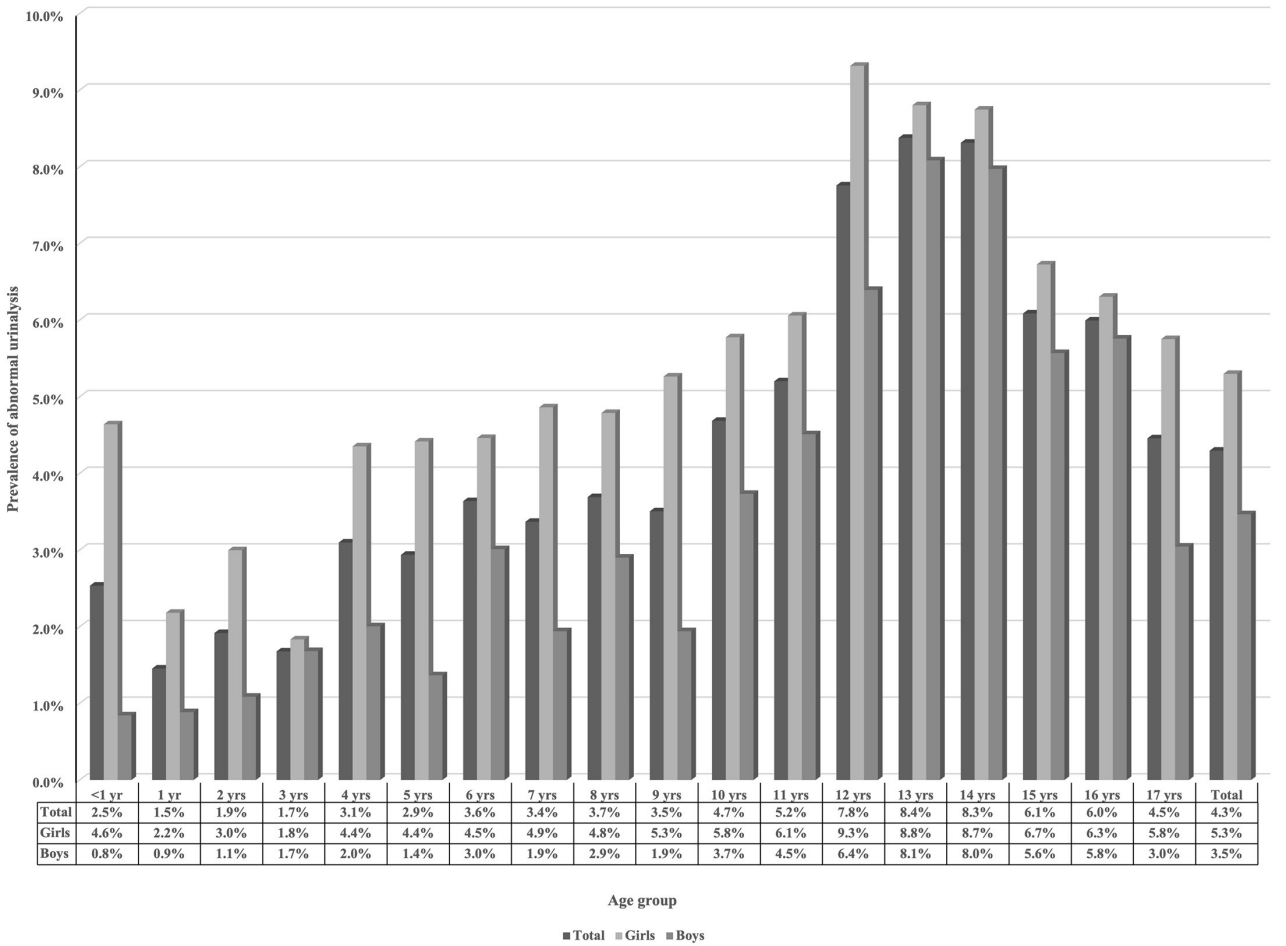
Age and sex distributions of abnormal urinalysis results in children.

The prevalence of abnormal urinalysis increased stepwise with increasing age, reaching a peak value at the ages of 12–14 years old. Abnormal urinalysis results were more prevalent among girls in any age group (Figure 2). Furthermore, isolated hematuria and isolated leukocyturia were more prevalent in girls. However, isolated proteinuria was more commonly observed in boys (Table 2).

**Table 2:** Patterns of abnormal urinalysis in children.

**Age group**	**Isolated hematuria**			**Isolated proteinuria**			**Hematuria and proteinuria**			**Isolated leukocyturia**		
	**Boys**	**Girls**	**Total**	**Boys**	**Girls**	**Total**	**Boys**	**Girls**	**Total**	**Boys**	**Girls**	**Total**
<1 year old	0.0%	0.3%	0.1%	0.7%	1.0%	0.8%	0.0%	0.2%	0.1%	0.0%	3.0%	1.3%
1 year old	0.3%	0.6%	0.4%	0.5%	0.5%	0.5%	0.0%	0.1%	0.0%	0.1%	0.8%	0.4%
2 years old	0.5%	0.9%	0.7%	0.5%	0.7%	0.6%	0.1%	0.0%	0.0%	0.1%	0.8%	0.4%
3 years old	1.2%	0.8%	0.9%	0.3%	0.5%	0.4%	0.1%	0.0%	0.0%	0.1%	0.3%	0.2%
4 years old	0.9%	1.5%	1.2%	0.9%	0.7%	0.8%	0.0%	0.0%	0.0%	0.1%	1.7%	0.9%
5 years old	0.3%	1.1%	0.7%	0.9%	0.5%	0.6%	0.1%	0.1%	0.1%	0.0%	2.3%	1.3%
6 years old	1.3%	1.7%	1.5%	1.0%	1.4%	1.2%	0.2%	0.1%	0.1%	0.1%	1.0%	0.6%
7 years old	1.0%	1.3%	1.1%	0.7%	0.6%	0.6%	0.2%	0.3%	0.2%	0.0%	2.0%	1.1%
8 years old	1.3%	1.2%	1.2%	1.3%	0.8%	1.0%	0.0%	0.3%	0.1%	0.2%	1.9%	1.0%
9 years old	0.8%	2.1%	1.4%	1.1%	0.9%	0.9%	0.1%	0.3%	0.2%	0.0%	1.7%	0.9%
10 years old	1.7%	1.8%	1.7%	1.9%	1.0%	1.4%	0.1%	0.6%	0.4%	0.1%	1.4%	0.7%
11 years old	1.3%	2.9%	2.0%	2.5%	1.4%	2.0%	0.4%	0.4%	0.4%	0.1%	0.8%	0.4%
12 years old	1.9%	3.2%	2.6%	3.3%	2.4%	2.9%	0.5%	0.7%	0.6%	0.2%	1.6%	0.9%
13 years old	1.8%	2.7%	2.3%	4.1%	3.4%	3.7%	1.0%	0.5%	0.7%	0.4%	1.6%	1.0%
14 years old	1.2%	2.0%	1.6%	4.8%	4.1%	4.4%	1.4%	0.4%	0.9%	0.0%	1.5%	0.7%
15 years old	1.0%	2.7%	1.9%	2.9%	1.9%	2.3%	0.4%	0.3%	0.3%	0.3%	1.6%	1.0%
16 years old	1.3%	1.8%	1.5%	3.0%	1.9%	2.3%	0.1%	0.3%	0.2%	0.7%	1.9%	1.4%
17 years old	1.3%	2.1%	1.7%	0.8%	1.3%	1.0%	0.3%	0.5%	0.4%	0.3%	1.1%	0.7%
All ages	1.1%	1.7%	1.4%	1.7%	1.3%	1.5%	0.3%	0.3%	0.3%	0.1%	1.5%	0.8%

Binary logistic regression was performed to identify risk factors for abnormal urinalysis (Table 3). Girls were 2.0 times (95% CI 1.8–2.3, *p* < 0.001) more likely to exhibit abnormal results than boys. Increasing age was significantly associated with a higher likelihood of abnormal urinalysis (*p* < 0.001). Compared with children whose guardians had a college degree or higher, those whose guardians had high school/technical secondary school (1.2, 1.1–1.4; *p* = 0.009) or lower than high school education (1.3, 1.0–1.5; *p* = 0.017) had a higher likelihood of abnormal urinalysis results after multivariable adjustment. Regarding geographic distribution, children from the southern region of China were least likely to present with urinary abnormalities (*p* < 0.001).

**Table 3:** Factors associated with abnormal urinalysis in children.

	**Univariate**		**Multivariable-adjusted**	
	**Odds ratio (95% CI)**	***P*-value**	**Odds ratio (95% CI)**	***P*-value**
Sex (girls vs. boys)	2.0 (1.9–2.3)	<0.001	2.0 (1.8–2.3)	<0.001
Age (per year old)	1.1 (1.1–1.1)	<0.001	1.1 (1.1–1.1)	<0.001
Guardian's education level		0.422		0.019
College degree or more	1.0 (reference)		1.0 (reference)	
Junior college degree	1.0 (0.8–1.1)	0.484	1.0 (0.9–1.2)	0.923
High school or technical secondary school	1.1 (0.9–1.2)	0.289	1.2 (1.1–1.4)	0.009
Less than high school	1.0 (0.9–1.2)	0.614	1.3 (1.0–1.5)	0.017
Geographic location		<0.001		<0.001
Guangzhou (south)	1.0 (reference)		1.0 (reference)	
Chengdu (southwest)	1.9 (1.6–2.2)	<0.001	2.1(1.7–2.5)	<0.001
Hangzhou (east)	3.1 (2.6–3.7)	<0.001	3.3 (2.7–4.1)	<0.001
Beijing (north)	2.1 (1.8–2.5)	<0.001	2.4 (2.0–2.9)	<0.001
Shenyang (northeast)	1.9 (1.6–2.2)	<0.001	2.2 (1.8–2.6)	<0.001
Monthly household income		0.014		0.308
<5,000 CNY	1.0 (reference)		1.0 (reference)	
5,000–10,000 CNY	1.0 (0.9–1.1)	0.470	1.1 (0.9–1.3)	0.328
>10,000 CNY	1.1 (0.9–1.3)	0.531	1.2 (1.0–1.5)	0.063
Refused to answer	0.8 (0.7–0.9)	0.003	1.1 (0.9–1.2)	0.563
Awareness of kidney diseases (poor vs. good)	1.2 (1.1–1.3)	<0.001	1.0 (0.9–1.1)	0.845
Attitudes toward routine urinalysis in children (necessary vs. others)^a^	1.1 (1.0–1.2)	0.031	1.0 (0.9–1.2)	0.565
Family size (>3 vs. ≤ 3 family members)	0.9 (0.8–1.0)	0.081	1.1 (1.0–1.2)	0.210

a *other attitudes toward routine urinalysis included “not necessary at all,” “it doesn't matter” or “I have no idea”*.

### Guardians' Attitudes, Awareness, and Compliance

Only 48.8% of the guardians agreed that routine urinalysis was necessary for children. A considerable number of the guardians presented with poor awareness of kidney diseases: 20.4% of the guardians did not know kidney diseases were harmful to children, and 41.8% had no idea about the signs of kidney diseases in children. Moreover, 17.7% of the guardians considered medications ineffective, unnecessary or preferred folk prescriptions for childhood kidney diseases.

Binary logistic regression was conducted to evaluate the impact of age, sex, geographic location, socioeconomic status, guardians' awareness or attitudes on participants' responsiveness. Girls were 1.3 times (95% CI 1.2–1.4, *p* < 0.001) more likely to respond to the second urinalysis than boys. Increasing age was associated with a lower response rate (*p* < 0.001). Children whose guardians considered routine urinalysis as necessary were 1.2 times (95% CI 1.0–1.3, *p* = 0.005) more likely to be responsive than others. Children from the southern region of China were most likely to respond (*p* < 0.001) after multivariable adjustment.

## Discussion

In our study, urinalysis screening was performed for more than 70,000 children of all ages in 5 cities in China, with diverse characteristics. The prevalence of abnormal urinalysis was estimated to be 4.3% in “healthy” children. In previous reports, the prevalence of abnormal results in children ranged from 0.5 to 5.5% (6, 8, 15–21). Patterns of abnormalities were variable according to different countries, age groups or screening methods. As reported by Japan, Egypt, China and Lebanon, isolated hematuria was the most prevalent (0.36–3.15%) (6, 15, 20, 21). However, in other reports from Malaysia and China (18, 19), proteinuria was more prevalent (1.85 and 0.51%). Most of the above studies screened school children by urine dipstick testing occult blood and protein. A recent investigation in Vietnam screened children aged 3–5 years old using a dipstick with 10 reagents (16). They reported that nitrituria (2%) and leukocyturia (1%) were more common. In our study, we screened children of all ages by dipstick analysis with 10 reagents. Isolated proteinuria (1.5%) was the most prevalent, followed by isolated hematuria (1.4%), isolated leukocyturia (0.8%), hematuria and proteinuria (0.3%). Sex and age differences in abnormality patterns were also observed.

Notably, among children initially detected as abnormal, only 30.8% of them remained abnormal on the second urinalysis. Poor consistency has also been observed by previous studies. Among children detected as abnormal by the first analysis, only 15–55% were confirmed abnormal after the second analysis (6, 17–21). This implied that more than half of the abnormalities by the first analysis might be transient or false positive. Furthermore, among “abnormal” children confirmed after the second urinalysis, more than 70% were excluded from having kidney diseases upon further examination (7, 21). Therefore, the cost-effectiveness of childhood urinalysis screening would be largely impaired due to poor reproducibility.

To improve the specificity and cost-effectiveness of urinalysis screening, a targeted screening strategy has been suggested among high-risk adults with diabetes mellitus, hypertension, cardiovascular disease or low socioeconomic status (13, 14). Unfortunately, there is not yet a well-defined target population in children, owing to limited evidence. Reported by Japan, the percentage of abnormal urinalysis in children aged 12–14 was higher than that in children aged 6–11 (6). In a Lebanese study of children aged 5–8 years old, positive results were shown to be more common among 6-year-old children (15). Hematuria and proteinuria were mainly detected in children from poor socioeconomic regions. An Egyptian study of children aged 6–13 years demonstrated that age, sex or socioeconomic status had no impact on the prevalence of urinary abnormalities (20). Heretofore, without a comprehensive investigation of potential risk factors among children of all ages, we are unable to define the target populations.

In this study, we performed urinalyses among children of all ages and investigated potential risk factors. Sex, age, guardian's education level and geographic location were observed to be significantly associated with abnormal urinalysis results. The prevalence of abnormalities was higher in girls of any age. The highest prevalence was observed in children aged 12–14, exactly the same as Japan reported (6). Interestingly, two steep rises in prevalence were noticed in children aged 4 and 12. Abnormal results were less common in the southern and southwestern regions of China, which had very high population densities. This is consistent with a previous report from Vietnam in which abnormal findings were more common in communities with a very low population density (16). Therefore, sex, age, guardian's educational level, and geographic location were revealed to be significantly associated with abnormal urinalysis. The associations might be owed to variable lifestyles, environments, health care resources or underlying diseases.

There were some limitations in the current study. First, 45% of the children with abnormal results in the first analyses did not respond to the second urinalyses. Potential non-response bias should be considered when interpreting the analysis results. Second, our study mainly focused on children from large cities. In the future, the prevalence and risk factors for abnormal urinalysis should also be investigated among children in rural or other areas. Third, this study did not include physical examinations such as height, weight and blood pressure. Some special high-risk groups of children, such as children with obesity or hypertension, should also be studied and considered in a screening approach. Fourth, in children not toilet trained, urine bags were used to collect urine samples. This might result in false-positive results. Finally, children with abnormal urinalysis still need further examination for the diagnosis of kidney diseases. It remained far from defining risk groups associated with diseases. Nonetheless, the current study investigated risk factors associated with abnormal urinalysis, which might benefit further practice.

In summary, we performed a large-scale, cross-sectional, multicenter study of children from infants to 17 years old. More than 70,000 children were randomly selected. The prevalence of abnormal urinalysis results was 4.3% in our study. To increase efficiency of the screening process, we suggest targeted screening. Girls, children aged 12–14 years old, and children whose guardians had a low educational level and children in certain geographic locations were high-risk populations that were significantly associated with abnormal urinalysis. Future prospective studies are still needed to compare the cost-effectiveness of different screening programs.

## Data Availability Statement

The raw data supporting the conclusions of this article will be made available by the authors, without undue reservation.

## Ethics Statement

The studies involving human participants were reviewed and approved by Ethics Committee of Peking University First Hospital. Written informed consent to participate in this study was provided by the participants' legal guardian/next of kin.

## Author Contributions

JD conceptualized and designed the study and critically reviewed and revised the manuscript. XZ designed the study, coordinated data collection, carried out the analyses, and drafted the initial manuscript. ZW, YG, YW, YS, HS, ZZ, XC, PZ, GX, and CY participated in the study design, supervised data collection, and reviewed and revised the manuscript. HZ, FZ, YT, HW, WW, WL, and WZ coordinated the data collection and reviewed and revised the manuscript. SZ and MS participated in the study design, carried out the analyses, and reviewed and revised the manuscript. All authors approved the final manuscript as submitted and agreed to be accountable for all aspects of the work.

## Conflict of Interest

The authors declare that the research was conducted in the absence of any commercial or financial relationships that could be construed as a potential conflict of interest.
